# A Silurian ancestral scorpion with fossilised internal anatomy illustrating a pathway to arachnid terrestrialisation

**DOI:** 10.1038/s41598-019-56010-z

**Published:** 2020-01-16

**Authors:** Andrew J. Wendruff, Loren E. Babcock, Christian S. Wirkner, Joanne Kluessendorf, Donald G. Mikulic

**Affiliations:** 1 Department of Biology and Earth Science, Otterbein University, Westerville, Ohio 43081 USA; 2 School of Earth Sciences, The Ohio State University, Columbus, Ohio 43210 USA; 3 Allgemeine & Spezielle Zoologie, Universität Rostock, Universitätsplatz 2, D-18055 Rostock, Germany; 4 Weis Earth Science Museum, University of Wisconsin-Fox Valley, Menasha, Wisconsin 54952 USA

**Keywords:** Palaeoecology, Evolutionary developmental biology, Palaeontology, Taxonomy, Palaeontology

## Abstract

Scorpions are among the first animals to have become fully terrestrialised. Their early fossil record is limited, and fundamental questions, including how and when they adapted to life on land, have been difficult to answer. Here we describe a new exceptionally preserved fossil scorpion from the Waukesha Biota (early Silurian, ca. 437.5–436.5 Ma) of Wisconsin, USA. This is the earliest scorpion yet reported, and it shows a combination of primitive marine chelicerate and derived arachnid characteristics. Elements of the circulatory, respiratory, and digestive systems are preserved, and they are essentially indistinguishable from those of present-day scorpions but share similarities with marine relatives. At this early point in arachnid evolution, physiological changes concomitant with the marine-to-terrestrial transition must have occurred but, remarkably, structural change in the circulatory or respiratory systems appear negligible. Whereas there is no unambiguous evidence that this early scorpion was terrestrial, this evidence suggests that ancestral scorpions were likely capable of forays onto land, a behavior similar to that of extant horseshoe crabs.

## Introduction

Scorpions include some of the earliest animals to have become fully terrestrial^1,2^. The earliest forms are Silurian in age^3,4^, but because most Paleozoic species are represented by only rare, fragmentary material, much of their early evolutionary history is speculative. Divergent views regarding the habitat of Paleozoic scorpions have been published. Some have argued that the earliest scorpions were marine^5,6^, whereas others have claimed a terrestrial origin^2,7^. It has also been argued that some Paleozoic scorpions were secondarily aquatic^8^. Arguments concerning whether early scorpions were terrestrial or marine have been based principally on stratigraphic context^9^ and preserved anatomical features. Anatomical evidence used to interpret habitat include the presence or absence of feeding structures used to liquify prey (coxapophyses or stomathecae)^10^, chemosensory organs (pectines)^11^, mechanosensory organs (trichobothria)^10^, and respiratory structures (book gills or book lungs)^7,8,11^. In some Paleozoic species, inferred locomotory stance, deduced from limb morphology^6^, also has been applied as evidence of habitat. All Silurian scorpion fossils, and indeed most from the Paleozoic, have been recovered from nearshore to marginal-marine strata. Such deposits provide ambiguous evidence of habitat, as bodily remains of arthropods are easily transported after death^12^. Likewise, inferring locomotory stance and habitat from limb morphology in a fossil scorpion can yield equivocal results^9^. Pectines, stomathecae, trichobothria, and respiratory structures are rarely preserved as fossils, and in many examples the non-preservation of such structures likely has a taphonomic origin^13,14^.

**Figure 1:**
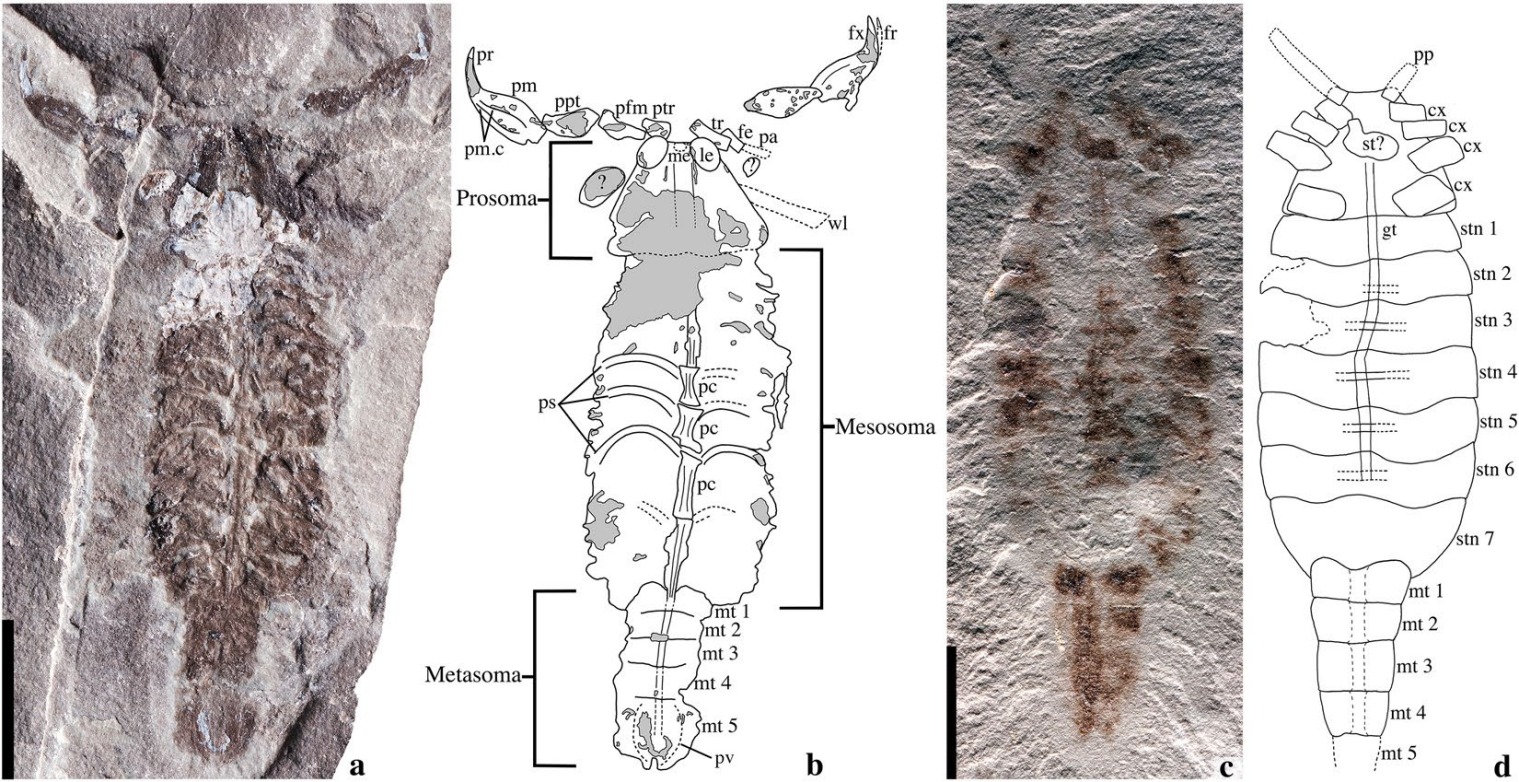
*Parioscorpio venator* gen. et sp. nov., Brandon Bridge Formation (Silurian), Wisconsin, USA. (**a**) Holotype, UWGM 2162, photographed under low-angle lighting and revealing internal anatomy; (**b**) interpretive drawing of holotype; (**c**) Paratype, UWGM 2163, photographed under low-angle lighting; (**d**) interpretive drawing of paratype. Abbreviations: cx, coxa; fe, femur; fr, free finger; fx, fixed finger; gt, gut; le, lateral eye; me, median eyes; mt, metasomal segment; pa, patella; pc, pericardium; pfm, pedipalp femur; pm, pedipalp manus; pm.c, pedipalp manus carina; ppt, pedipalp patella; pr, pedipalp ramus; ps, pulmo-pericardial sinus; ptr, pedipalp trochanter; pv, poison vesicle; st, sternum; stn, sternite; tr, trochanter; wl, walking leg. Scale bar equals 5 mm.

**Figure 2:**
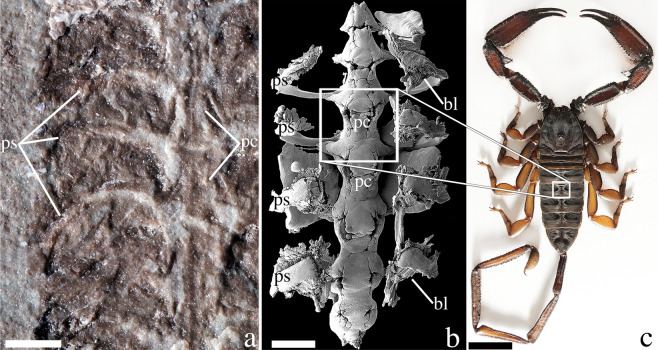
Medial structures associated with the pulmonary-cardiovascular system in Silurian (**a**) and Holocene (**b**,**c**) scorpions. (**a**) *Parioscorpio venator* gen. et sp. nov., holotype, detail of medial region showing pulmo-cardiovascular structures; (**b**) SEM of *Centruroides exilicauda*, corrosion cast of pericardium and associated pulmo-pericardial sinuses; (**c**) *Hadogenes troglodytes*, male, dorsal surface, showing medial structures externally reflecting the position of the internal pericardium (compare with B). Abbreviations: bl, book lungs; pc, pericardium; ps, pulmo-pericardial sinus. Scale bars equal 1 mm for (**a,b**); scale bar equals 1 cm for (**c**).

**Figure 3:**
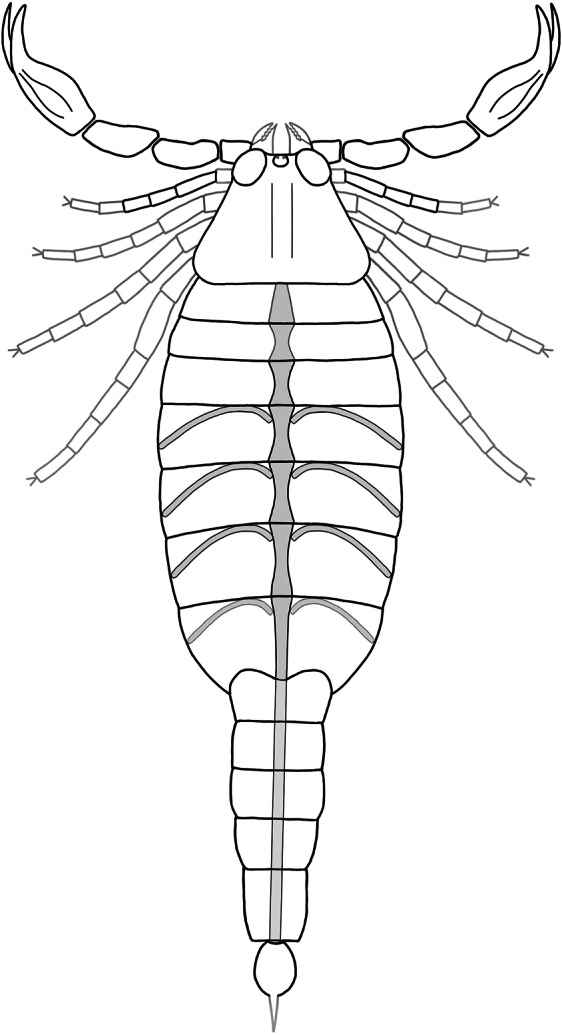
Reconstruction of *Parioscorpio venator* gen. et sp. nov. Structures outlined in grey are inferred based on *Proscorpius osborni*. Structures highlighted with grey infilling are the preserved elements of the pulmonary-cardiovascular system.

Here we report exceptionally preserved remains of a new Silurian scorpion, *Parioscorpio venator* gen. et sp. nov. (Figs. 1, 2a, 3, Supplementary Figs. 1–3). It occurs in strata that are older than those from Scotland yielding *Dolichophonus loudonensis*^15^, which was previously accepted as the oldest known scorpion^16^. The new species shows some primitive features, which support an interpretation of this animal close to the base of the arachnid clade. It also shows some derived features indicative of scorpions. Internal anatomy, including parts of the respiratory, circulatory (vascular and lacunar) and digestive systems (Figs. 1, 2a, Supplementary Fig. 1), has been preserved, and provides insight into a group that terrestrialised early in its evolutionary history.

## Material and Stratigraphic Context

Study material consists of two substantially complete specimens (Fig. 1, Supplementary Figs. 1–3) from the Waukesha Lagerstätte in the lower Brandon Bridge Formation near Waukesha, Wisconsin, USA^17–21^. Remains are preserved as a combination of thin organic film and replication by thin calcium phosphate coating. Associated graptolites^17–21^ and conodont elements^20^ indicate an early Silurian age (Llandovery Series, Telychian Stage). Recent biostratigraphic assessment of the Waukesha Lagerstätte^22^ shows that conodonts present include *Pterospathodus eopennatus*, the eponymous indicator of the *P. eopennatus* Superzone (Telychian Stage; c. 437.5–436.5 Ma).

The Waukesha Biota is diverse^17,18,20,21^, and includes biomineralised and non-biomineralising or lightly skeletonised taxa, of which macrofossils are assigned to at least 12 metazoan phyla. Articulated trilobites, ostracodes, conulariids, graptolites, non-biomineralised arthropods and ‘worms’ dominate the biota. Atypical marine conditions are suggested by the near lack of echinoderm, brachiopod, mollusk and coral remains. Fossils are preserved in a 12 cm layer of thinly laminated, fine-grained dolostone deposited during a transgressive episode over the eroded dolostones of the Schoolcraft and Burnt Bluff formations^23^.

Fossils of *Parioscorpio venator* gen. et sp. nov. are from shallow marine sediments that accumulated in a sedimentary trap at the toe of an erosional scarp. The Schoolcraft and Burnt Bluff formations were subaerially exposed prior to the time of Brandon Bridge sedimentation, creating an 8-m scarp with a gentle slope and epikarstic features^19,23^. Sedimentary traps which formed along the base of the scarp in the intertidal or supratidal zones received remains of organisms washed in from nearby areas. These sedimentary traps have so far not yielded remains of unambiguous, fully terrestrialised animals or plants. Organisms were preserved through replication in microbial mats which coated and cemented bodily remains in place. Microbial processes are implicated in the precipitation of thin phosphatic coatings on non-biomineralised or lightly skeletonised remains leading to exceptional preservation^20,21,24^.

## Results

### Systematic Palaeontology

**Order** Scorpiones Koch, 1837.

**Family** Undetermined.

**Genus** *Parioscorpio* gen. nov.

**Etymology**. From Latin, *pario*, progenitor, and *scorpio*, scorpion.

**Type Species**. *Parioscorpio venator* sp. nov.

**Diagnosis**. As for *P. venator*, see below.

**Distribution**. Silurian (Llandovery, Telychian; c. 437.5-436.5 Ma), Wisconsin, USA.

***Parioscorpio venator*** **gen. et sp. nov**. Figures 1, 2a and 3).

**Etymology**. From Latin, *venator*, hunter.

**Types**. Holotype, University of Wisconsin Geology Museum, Madison, Wisconsin, UWGM 2162. Paratype, UWGM 2163.

**Location**. Waukesha Lime and Stone Company west quarry, north of State Highway 164, Waukesha, Wisconsin, USA.

**Horizon**. Lower part of the Brandon Bridge Formation (Silurian: Llandovery, Telychian).

**Diagnosis**. Prosoma subtrapezoidal with large eyes situated anterolaterally and ocelli situated anteromedially; pedipalps large, with tibia (fixed finger) elongate, swollen proximally in manus, narrow and recurved distally in ramus; mesosoma moderately wide and much longer than the metasoma, containing 7 dorsal tergites and 7 ventral sternites; sternites 1–2 short (sagitally), length increasing posteriorly. Metasoma excluding telson, approximately 1/3 length of opisthosoma, containing five narrow, subequal, weakly bilobate segments. Telson swollen proximally.

**Discussion**. *Parioscorpio venator* gen. et sp. nov. is now the earliest known scorpion. Conodont biostratigraphic zonation^22^ places the Waukesha Lagerstätte in the *Pterospathodus eopennatus* Superzone (Telychian Stage; c. 437.5–436.5 Ma). *Dolichophonus loudonensis* from the Eurypterid Bed (Deerhope Formation) at Gutterford Burn in the Pentland Hills, Scotland^15^ was previously the earliest described scorpion. Specimens of *D*. *loudonensis* are found in association with conodonts assigned to *Pterospathodus amorphognathoides* and graptolites indicative of the middle *Oktavites spiralis* Zone to the middle *Cyrtograptus lapworthi* Zone^25,26^. Together, conodont and graptolite evidence shows that *D*. *loudonensis* occurs in strata correlative with the *P. amorphognathoides* Zone (*P. celloni* Superzone; Telychian Stage, c. 435.5–434.5 Ma), which overlies the *P. eopennatus* Superzone.

**Figure 4:**
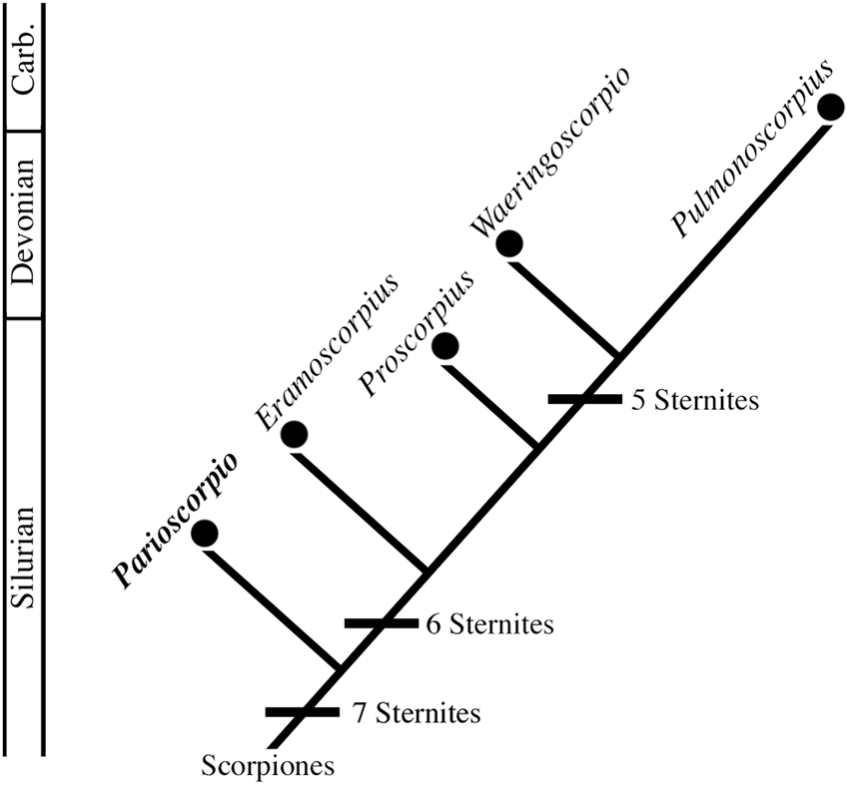
Hypothesis of relationship based on character transformation (number of sternites) among some of the more completely known Paleozoic scorpions, with geologic time scale at left. One important trend in the evolution of early scorpions is a reduction in the number of sternites. *P. venator* shows the most primitive condition known, seven sternites. There was progressive reduction to six and eventually five sternites.

*Parioscorpio venator* gen. et sp. nov. is characterised by a small exoskeleton showing a unique array of characters. Based on earlier studies^3,5,27–32^ some of these characters, such as compound eyes, are primitive (plesiomorphic) for arachnids. Other characters, such as clawed pedipalps and a narrow metasoma terminating in a stinger, are derived (apomorphic). A mesosoma container seven tergites and sternites (Fig. 1c,d), which is observed uniquely in *P. venator*, is interpreted as a primitive characteristic (Fig. 4). Paleozoic scorpions show a trend toward reducing the number of sternites through time. Six sternites are present in two Silurian species that are younger, *Proscorpius osborni*^28^ and *Eramoscorpius brucensis*^6^. Most extant and extinct scorpions have five sternites^29,30^, a condition that had evolved by at least the Carboniferous Period. The large, anterolateral eyes, and anteromedial position of the small medial eyes, also are regarded as plesiomorphic features, as they are present in younger Silurian species such as *Allopalaeophonus caledonicus*^31^, *Palaeophonus* nuncius^32^, and *Proscorpius osborni*^28^. Pectines, which are chemosensory structures present in all extant scorpions, are unknown in most Paleozoic forms including *P. venator*. This is probably a taphonomic artefact, as pectines are easily lost after death or moulting^13,14^. In other aspects of external morphology *P. venator* fits within the range of morphological features exhibited among other, more derived, scorpion taxa. A telson bearing an expanded area for a poison vesicle and a stinger is an apomorphous condition for scorpions^27^. The holotype of *P. venator* preserves an incomplete telson, which is folded under the fifth metasomal segment. The proximal portion shows a swelling close to the articulation with the metasoma, inferred to be a poison vesicle, but the more terminal stinger is not evident.

Both specimens of *P. venator* show details of internal anatomy. In the paratype, parting of the rock through the fossil reveals a medial structure interpreted as the gut tract (Fig. 1c,d), and it resembles that of extant scorpions. It is a narrow, simple tube extending from the prosoma to the metasoma. It originates in the anterior prosoma, slightly forward of the inferred position of the mouth.

In the holotype of *P. venator*, internal structures of the mesosoma and metasoma have been impressed on the thin dorsal cuticle during sediment compaction. They consist of a series of narrow, hourglass-shaped medial structures extending much of the length of the mesosoma (Fig. 1a,b, Supplementary Fig. 1e). Extending laterally from each of the medial structures is a pair of curved, strut-like elements (Fig. 1a,b, Supplementary Fig. 1d). The hourglass-shaped structures of the mesosoma continue into the metasoma as a simple, narrow tube. Neither book lungs nor book gills are evident on the fossil.

Detailed studies of the central architecture of the circulatory and respiratory systems in present-day scorpions^33,34^ reveal a strikingly similar arrangement to the preserved structures in *P. venator* (Figs. 1a,b, 2a). The pericardium, which surrounds and houses the heart, comprises a series of narrow, medial hourglass-shaped structures in the mesosoma. Strut-like pulmo-pericardial sinuses project laterally from the pericardium (Fig. 2). In some extant scorpions, these internal medial structures are reflected externally on the dorsal cuticle (Fig. 2c), along with the tergite boundaries. This is evident on *P. venator* as well. In extant scorpions, the pulmo-pericardial sinuses connect the book lungs with the circulatory system. The book lungs oxygenate the hemolymph (‘blood’) and deliver the oxygenated hemolymph to the pericardium^33,34^. We infer that the organs of the respiratory-cardiovascular architecture were evolutionarily conservative.

Among extant chelicerates, terrestrial forms such as scorpions tend to be restricted to processing oxygen from air (e.g., by means of book lungs). However, marine xiphosurans, which normally extract oxygen from water by means of external book gills, are nevertheless capable of respiration when they journey onto land to spawn^35^. The circulatory and respiratory organs of xiphosurans (horseshoe crabs) are equally complex to those of scorpions^34,36^, and this may contribute to their ability to respire in air and survive on land. Presumably, ancient xiphosurans and arachnid ancestors had a similar capability to venture onto land.

Anatomical details preserved in *P. venator* suggest that the physiological changes necessary to accommodate a marine-to-terrestrial transition in arachnids occurred early in their evolutionary history. Whether *P. venator* was a fully terrestrial arthropod is uncertain. The close similarity of its preserved pulmonary-cardiovascular structures with those of extant scorpions and horseshoe crabs hint at the possibility of extended stays on land.

## Methods

All specimens figured and discussed here are held by the University of Wisconsin-Madison Geology Museum, Madison, Wisconsin, USA. Specimens photographed using a Canon EOS Rebel T3i Digital SLR with a Canon MP-E 65 mm macro lens and full spectrum lighting. Some images were made using low-angle lighting. Images were z-stacked and stitched using Adobe Photoshop CC. A corrosion cast of the pericardium and pulmo-pericardial sinuses of a present-day scorpion (*Centruroides exilicauda*) was used for anatomical comparison. Another present-day scorpion (*Hadogenes troglodytes*) was used for morphological comparison of medial structures. Explanatory diagrams and the reconstruction of *Parioscorpio venator* were created using Microsoft Surface Pro 3 with a stylus using Photoshop CC.

## Supplementary information

Supplementary Figures

## References

[1] Dunlop, J. A., Scholtz, G. & Selden, P. A. Water-to-land transitions in *Arthropod Biology and Evolution*. (eds. Minelli, A., Boxshall, G. & Fusco, G. 417–439 (Springer, 2013).

[2] Dunlop JA, Webster M (1999). Fossil evidence, terrestrialization and arachnid phylogeny. J. Arachn..

[3] Selden, P. A. & Dunlop, J. A. Fossil taxa and relationships of chelicerates in *Arthropod Fossils and Phylogeny*. (ed, Edgecombe, G. D.) 303–332 (Columbia University Press, 1998).

[4] Anderson LI, Clarkson ENK, Steward SE, Mitchell D (2007). An upper Llandovery Konservat-Lagerstätte in a depositional context: the Pentland Hills Eurypterid Bed, Midlothian. Scot. J. Geol..

[5] Jeram AJ (1998). Phylogeny, classification and evolution of Silurian and Devonian scorpions. Proc. 17th Eur. Colloq. Arachn. Edinb..

[6] Waddington J, Rudkin DM, Dunlop JA (2015). A new mid-Silurian aquatic scorpion–one step closer to land?. Biol. Lett..

[7] Kühl G, Bergmann A, Dunlop JA, Garwood RJ, Rust J (2012). Redescription and palaeobiology of *Palaeoscorpius devonicus* Lehman, 1944 from the Lower Devonian Hunsrück Slate of Germany. Palaeontology.

[8] Poschmann M, Dunlop JA, Kamenz C, Scholtz G (2008). The Lower Devonian scorpion *Waeringoscorpio* and the respiratory nature of its filamentous structures, with the description of a new species from the Westerwald area, Germany. Paläont. Z..

[9] Selden PA, Jeram AJ (1989). Palaeophysiology of terrestrialisation in Chelicerata. Trans. R. Soc. Edinb..

[10] Legg DA, Garwood RJ, Dunlop JA, Sutton MD (2012). A taxonomic revision of orthosternous scorpions from the English Coal Measures aided by x-ray micro-tomography (XMT). Palaeontol. Electron..

[11] Jeram AJ (1994). Scorpions from the Viséan of East Kirkton, West Lothian, Scotland, with a revision of the infraorder Mesoscorpionina. Trans. R. Soc. Edinb..

[12] Babcock LE, Merriam DF, West RR (2000). *Paleolimulus*, an early limuline (Xiphosurida), from Pennsylvanian–Permian Lagerstätten of Kansas and taphonomic comparison with modern *Limulus*. Lethaia.

[13] Scholtz G, Kamenz C (2006). The book lungs of Scorpiones and Tetrapulmonata (Chelicerata, Arachnida): Evidence for homology and a single terrestrialisation event of a common arachnid ancestor. Zoology.

[14] McCoy VE, Brandt DS (2009). Scorpion taphonomy: criteria for distinguishing fossil scorpion molts and carcasses. J. Arachn..

[15] Laurie M (1899). On a Silurian scorpion and some additional eurypterid remains from the Pentland Hills. Trans. R. Soc. Edin..

[16] Selden PA, Dunlop JA (2013). Scorpion fragments from the Silurian of Powys, Wales. Arachnol..

[17] Mikulic DG, Briggs DEG, Kluessendorf J (1985). A Silurian soft-bodied biota. Science.

[18] Mikulic DG, Briggs DEG, Kluessendorf J (1985). A new exceptionally preserved biota from the lower Silurian of Wisconsin, USA. Philos. Trans. R. Soc. London Ser. B..

[19] Kluessendorf J, Mikulic DG (1996). An early Silurian sequence boundary in Illinois and Wisconsin. Geol. Soc. Am, Sp, Paper.

[20] Wendruff A. J., Babcock, L. E., Mikulic, D. G. & Kluessendorf, J. Palaeobiology and Taphonomy of Exceptionally Preserved Organisms from the Waukesha Biota (Silurian), Wisconsin, USA. *Palaeogeogr., Palaeoclimatol., Palaeoecol*., in press.

[21] Babcock L. E., Wendruff, A. J., Mikulic, D. G. & Kluessendorf, J. Fossilized digestive tracts of arthropods and worms from the Waukesha Lagerstätte, Silurian of Wisconsin, USA. *Geol. Soc. Am. N-C. Meeting Abstr*. **48**(5), 10.1130/abs/2016NC-275201 (2016).

[22] Kleffner, M. A., Norby, R. D., Kluessendorf, J. & Mikulic, D. G. Revised conodont biostratigraphy of Lower Silurian strata of southeastern Wisconsin. *Geol. Soc. Am. N.-C. Meeting Abstr*. **50**(4), 10.1130/abs/2018NC-312921 (2018).

[23] Mikulic, D. G. & Kluessendorf, J. Sequence stratigraphy and depositional environments of the Silurian and Devonian rocks of Southeastern Wisconsin. *SEPM Great Lakes Section & Michigan Basin Geol. Soc*., Waukesha, Wisconsin, 1–84 (1998).

[24] Wendruff, A. J., Babcock, L. E., Kluessendorf, J. & Mikulic, D. G. The Waukesha Biota: an unusual glimpse of life on a Silurian Carbonate Platform. *Geol. Soc. Am. N.-C. Meeting Abstr*. **48**(5), 10.1130/abs/2016NC-275201 (2016).

[25] Bull EE, Loydell DK (1995). Uppermost Telychian graptolites from the North Esk Inlier, Pentland Hills, near Edinburgh. Scot. J. Geol..

[26] Loydell DK (2005). Graptolites from the Deerhope Formation, North Esk Inlier. Scott. J. Geol..

[27] Dunlop JA (2010). Geological history and phylogeny of Chelicerata. Arthropod Struct. Dev..

[28] Dunlop JA, Tetlie OE, Prendini L (2008). Reinterpretation of the Silurian scorpion *Proscorpius osborni* (Whitfield): integrating data from Palaeozoic and recent scorpions. Palaeontology.

[29] Hjelle, J. T. Anatomy and morphology in *Biology of Scorpions*. (ed. Polis, G. A.) 9–63 (Stanford University Press, 1990)

[30] Sissom, W. D. Systematics, biogeography and paleontology in *Biology of Scorpions*. (ed. Polis, G. A.) 64–160 (Stanford University Press, 1990).

[31] Petrunkevitch A (1953). Paleozoic and Mesozoic Arachnida of Europe. Geol. Soc. Am. Mem..

[32] Kjellesvig-Waering, E. N. *A Restudy of the Fossil Scorpionida of the World*. 1–287 (Paleontological Research Institution, 1986).

[33] Wirkner CS, Prendini L (2007). Comparative morphology of the hemolymph vascular system in scorpions–a survey using corrosion casting, MicroCT, and 3D reconstruction. J. Morphol..

[34] Wirkner, C. S., Tögel, M. & Pass, G. The arthropod circulatory system in *Arthropod Biology and Evolution* (eds. Minelli, A., Boxshall, G. & Fusco, G.) 343–391 (Springer, 2013).

[35] Bottom ML, Shuster CN, Sekiguchi K, Sugita H (1996). Amplexus and mating behavior in the Japanese horseshoe crab, *Tachypleus tridentatus*. Zool. Sci..

[36] Göpel T, Wirkner CS (2015). An “ancient” complexity? Evolutionary morphology of the circulatory system in Xiphosura. Zoology.

