# Author Correction: A Silurian ancestral scorpion with fossilised internal anatomy illustrating a pathway to arachnid terrestrialisation

**DOI:** 10.1038/s41598-020-77333-2

**Published:** 2020-11-18

**Authors:** Andrew J. Wendruff, Loren E. Babcock, Christian S. Wirkner, Joanne Kluessendorf, Donald G. Mikulic

**Affiliations:** 1 Department of Biology and Earth Science, Otterbein University, Westerville, Ohio 43081 USA; 2 School of Earth Sciences, The Ohio State University, Columbus, Ohio 43210 USA; 3 Allgemeine & Spezielle Zoologie, Universität Rostock, Universitätsplatz 2, 18055 Rostock, Germany; 4 Weis Earth Science Museum, University of Wisconsin-Fox Valley, Menasha, Wisconsin 54952 USA

Correction to: *Scientific Reports* 10.1038/s41598-019-56010-z, published online 16 January 2020

The original version of this Article was not registered at Zoobank prior to publication. The ‘Results’ section should contain the following:

“**Systematic Palaeontology**

**Order** Scorpiones Koch, 1837

**Family** Undetermined

**Genus** *Parioscorpio* gen. nov.

urn:lsid:zoobank.org:act:453B4AF9-8535-4671-8488-01C6A6D9EDB5

**Etymology.** From Latin, *pario*, progenitor, and *scorpio*, scorpion.

**Type Species.** *Parioscorpio venator* sp. nov.

**Diagnosis.** As for *P. venator*, see below.

**Distribution.** Silurian (Llandovery, Telychian; c. 437.5-436.5 Ma), Wisconsin, USA.

***Parioscorpio venator*** **gen. et sp. nov.** Figures 1, 2a and 3)

urn:lsid:zoobank.org:act:0FCB6DFC-3D95-4B5D-8751-16FF24FACF6C

**Etymology.** From Latin, *venator*, hunter.

**Types**. Holotype, University of Wisconsin Geology Museum, Madison, Wisconsin, UWGM 2162. Paratype, UWGM 2163.

**Location.** Waukesha Lime and Stone Company west quarry, north of State Highway 164, Waukesha, Wisconsin, USA.

**Horizon.** Lower part of the Brandon Bridge Formation (Silurian: Llandovery, Telychian).

**Diagnosis.** Prosoma subtrapezoidal with large eyes situated anterolaterally and ocelli situated anteromedially; pedipalps large, with tibia (fixed finger) elongate, swollen proximally in manus, narrow and recurved distally in ramus; mesosoma moderately wide and much longer than the metasoma, containing 7 dorsal tergites and 7 ventral sternites; sternites 1-2 short (sagitally), length increasing posteriorly. Metasoma excluding telson, approximately 1/3 length of opisthosoma, containing five narrow, subequal, weakly bilobate segments. Telson swollen proximally.

**Nomenclatural Acts**

The electronic edition of this article conforms to the requirements of the amended International Code of Zoological Nomenclature, and hence the new names contained herein are available under that Code from the electronic edition of this article. This published work and the nomenclatural acts it contains have been registered in ZooBank, the online registration system for the ICZN. The ZooBank LSIDs (Life Science Identifiers) can be resolved and the associated information viewed through any standard web browser by appending the LSID to the prefix “http://zoobank.org/”. The LSID for this publication is: urn:lsid:zoobank.org:pub:0098FF35-25DC-4CE9-8CAF-F952EE77A785. The electronic edition of this work was published in a journal with an ISSN, and has been archived and is available from the following digital repositories: PubMed Central, LOCKSS.”

