# Supplementary material for: A Silurian ancestral scorpion with fossilised internal anatomy illustrating a pathway to arachnid terrestrialisation
Source: Sci Rep. 2020 Jan 16;10:14. doi: 10.1038/s41598-019-56010-z (PMC6965631; doi:10.1038/s41598-019-56010-z)
Supplement: Supplementary file 1 — Supplementary Figures [file 41598_2019_56010_MOESM1_ESM.pdf]

A Silurian ancestral scorpion with fossilised internal anatomy illustrating a pathway to arachnid terrestrialisation

Andrew J. Wendruff[1], Loren E. Babcock[2], Christian S. Wirkner[3], Joanne Kluessendorf[4], and Donald G. Mikulic[4]

[1]Department of Biology and Earth Science, Otterbein University, Westerville, Ohio 43081, USA.

[2]School of Earth Sciences, The Ohio State University, Columbus, Ohio 43210, USA.

[3]Allgemeine & Spezielle Zoologie, Universität Rostock, Universitätsplatz 2, D-18055 Rostock, Germany. [4]Weis Earth Science Museum, University of Wisconsin-Fox Valley, Menasha, Wisconsin 54952, USA. Correspondence and requests for materials should be addressed to A.J.W. (email: wendruff1@otterbein.edu)

Supplementary Information

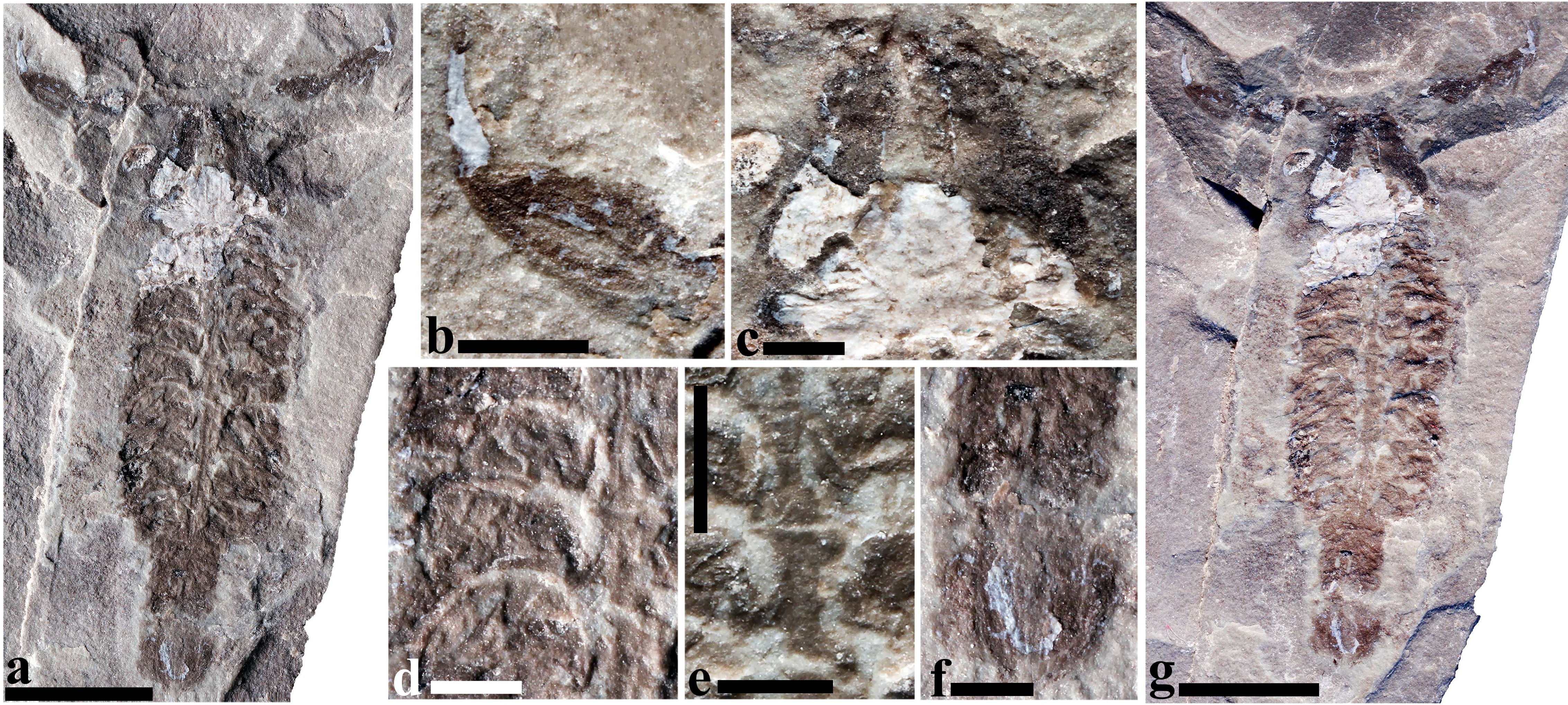

Supplementary Figure 1

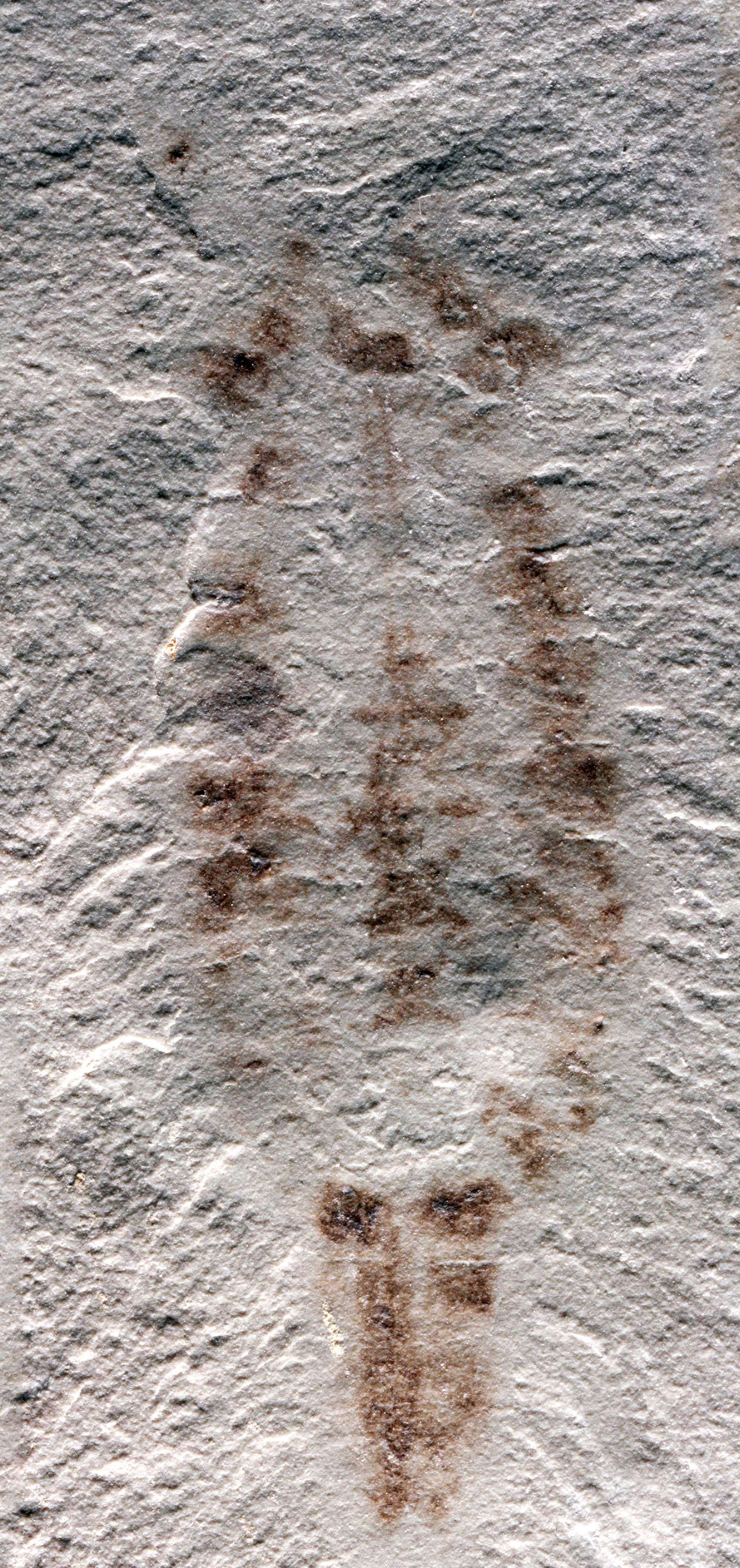

Supplementary Figure 2

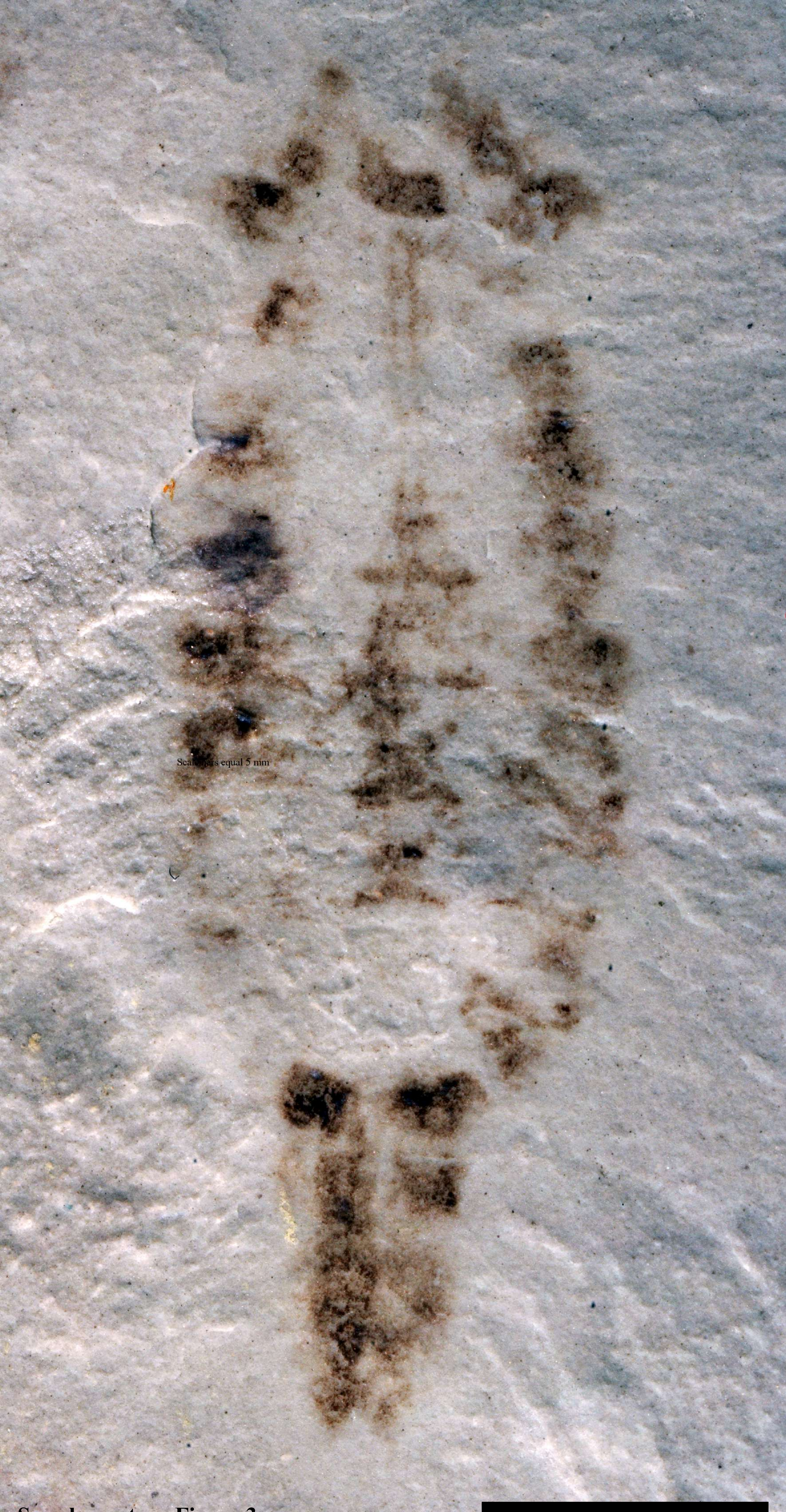

Scale bars equal 5 mm

Supplementary Figure 3

Supplementary Figure 1. Additional images of the holotype of *Parioscorpio venator* gen. et sp. nov. (UWGM 2162), Brandon Bridge Formation (Silurian), Wisconsin, USA. **a** entire specimen photographed under low-angle lighting and revealing internal anatomy; **b** closeup of left pedipalp; **c** closeup of prosoma showing the large, anterolateral eyes; **d** closeup of the strut-like pulmo-pericardial sinuses that project from the pericardium; **e** closeup of medial hourglassshaped pericardium; **f** closeup of end of the metasoma showing a bulbous vesicle folded over the previously metasomal segment, terminal stinger missing; **g** entire specimen photographed with diffuse lighting. Scale bars equal 5 mm for **a** and **g**; scale bar equals 1 mm for **b–f.**

Supplementary Figure 2. Additional image of the paratype of *Parioscorpio venator* gen. et sp. nov. (UWGM 2163), Brandon Bridge Formation (Silurian), Wisconsin, USA photographed under low angle lighting. Scale bars equal 5 mm.

Supplementary Figure 3. Additional image of the paratype of *Parioscorpio venator* gen. et sp. nov. (UWGM 2163), Brandon Bridge Formation (Silurian), Wisconsin, USA photographed wet under diffuse lighting. Scale bars equal 5 mm.
